# HLA‐DR Matching in Kidney Transplantation: Ethnic Disparities in Clinical Benefit and Policy Implications From a UK Registry Analysis

**DOI:** 10.1111/ctr.70429

**Published:** 2026-01-06

**Authors:** Hatem Ali, David Briggs, Nithya Krishnan

**Affiliations:** ^1^ Renal Department University Hospitals of Wales, Cardiff and Vale University Health Board Cardiff UK; ^2^ NHS Blood and Transplant Bristol UK; ^3^ Renal Department University Hospitals of Coventry and Warwickshire Coventry UK; ^4^ The Centre of Healthcare and Communities Coventry University Coventry UK

**Keywords:** alloimmunity, ethnic disparities, graft survival, HLA‐DR mismatch, kidney transplantation, UK Kidney Allocation Scheme

## Abstract

**Background:**

The UK Kidney Allocation Scheme (KAS) prioritizes organ allocation based on HLA mismatches, assigning the greatest weight to HLA‐DR compatibility. However, the clinical relevance of this approach across different ethnicities in the era of modern immunosuppression remains uncertain.

**Methods:**

We conducted a retrospective cohort study of 25 094 adult deceased donor kidney transplants in the United Kingdom between 2008 and 2020. Using competing risk Cox regression, we evaluated the impact of individual HLA locus mismatches and grouped mismatch levels (as defined by UK‐KAS) on graft survival. Subgroup analyses by ethnicity were performed, and the relationship between HLA mismatches and acute rejection was assessed using logistic regression.

**Results:**

A single HLA‐DR mismatch was significantly associated with graft failure (SHR 1.119, 95% CI 1.035–1.211, *p* = 0.005), while mismatches at the A, B, and DQ loci were not. In subgroup analyses, HLA‐DR mismatching was predictive of graft failure in Asian recipients but not in Black recipients. Black patients also exhibited higher rates of mismatching at all loci. DQ mismatches were associated with early acute rejection but did not predict long‐term graft failure. Ten‐year graft survival was 13% less with one HLA DR mismatch, and 17% less with 2 HLA DR mismatch, in comparison to zero DR mismatch. The four‐level HLA mismatch grouping used by UK‐KAS stratified risk incrementally, with levels 3 and 4 associated with 13% and 19% higher failure risk, respectively.

**Conclusions:**

HLA‐DR matching improves graft survival overall but offers limited benefit in Black recipients, likely due to low‐resolution typing inadequately capturing immunological compatibility across ethnic lines. The current UK‐KAS scoring system may inadvertently disadvantage ethnic minorities by delaying transplantation for matches that confer minimal benefit. Our findings support incorporating ethnicity‐specific considerations into kidney allocation policy to promote equity and optimize outcomes.

## 1 Introduction

The United Kingdom's Kidney Allocation Scheme (KAS) is designed to optimize organ allocation by assigning priority based on multiple factors, including Human Leukocyte Antigen (HLA) compatibility. Central to this framework is a scoring system that rewards lower mismatches at the HLA‐DR, HLA‐B, and HLA‐A loci, with DR mismatching carrying the greatest weight. This system, implemented during an era of limited immunosuppressive options, assumes that HLA matching universally improves transplant outcomes across all populations [1, 2, 3, 4].

However, modern immunosuppressive regimens—particularly the widespread use of tacrolimus and mycophenolate mofetil—have substantially reduced the impact of HLA mismatches on graft survival. In parallel, increasing ethnic diversity in transplant recipients has exposed limitations in the applicability of traditional matching algorithms. Notably, the reliance on low‐resolution HLA typing, as currently used in UK allocation, may mask important immunological differences between donor and recipient, especially in racially or ethnically discordant pairs. For example, a DR “match” in a Black recipient may not equate to a true molecular match when the donor is of European ancestry due to differences in underlying allele frequencies and linkage disequilibrium patterns [5, 6, 7].

This raises a critical question: Does DR matching offer equal clinical benefit to all ethnic groups? If not, current policies may unintentionally disadvantage ethnic minorities—particularly Black patients—by delaying access to transplantation for the sake of achieving DR matches that confer little or no survival advantage. This concern has been previously raised through imputation‐based studies suggesting that low‐resolution matching inadequately reflects high‐resolution compatibility, especially across ethnic lines [8]. Yet, real‐world outcome data demonstrating this discrepancy remain limited.

In this study, we conduct a comprehensive analysis of over 25 000 deceased donor kidney transplants in the United Kingdom between 2008 and 2020. We examine the association between HLA mismatches and graft survival, with a particular focus on ethnic differences in the clinical relevance of DR matching. By investigating whether the current allocation framework equitably translates HLA compatibility into clinical benefit across diverse populations, we aim to inform policy reform that ensures fairness, accuracy, and optimal use of scarce donor organs.

As a secondary objective, we also assess the predictive validity of the HLA mismatch groupings (levels 1–4) currently used in the UK KAS.

## 2 Methodology

### 2.1 Design and Study Cohort

All deceased donor adult kidney transplant patients registered in the UK Transplant registry database and had their transplant between 1st of January 2008 and 31st of December 2020 were retrospectively reviewed. We decided to recruit patients from 2008 onward as this period can reflect the current practice in transplant immunosuppression protocols which has been approximately stable since the publication of the SYMPHONY study in 2007 [9]. Furthermore, the molecular methods for HLA typing have been standardized since 2008 [10]. Exclusion criteria included patients with missing HLA mismatch, and patients with primary non‐functioning kidneys. Data collected were recipient characteristics (age, sex, BMI, ethnicity, history of diabetes, recipient CMV status, and dialysis before transplantation), donor characteristics (age, sex, ethnicity, CMV status, extended criteria donor, and donor hypertension), and transplant characteristics (cold ischemia time, delayed graft function, PRA, HLA‐A, B, DR, and DQ mismatch).

### 2.2 Main Outcomes and Definitions

The primary outcome measured was graft survival. Graft failure was defined as those with a low estimated glomerular filtration rate (eGFR) sufficient to be relisted for transplantation or return to dialysis. The outcome was censored at time of loss to follow‐up, end of follow‐up.

In addition to evaluating the individual effects of mismatches at each HLA locus, we also examined the predictive validity of the four‐level HLA mismatch grouping system currently used in the UK Kidney Allocation Scheme.

We also assessed the occurrence of acute rejection within 3 months post‐transplant and its relationship with different HLA mismatches.

### 2.3 Data Analysis

#### 2.3.1 Baseline Characteristics

To evaluate the baseline characteristics of the study population, we prepared a descriptive statistics table to show the distribution of variables among deceased kidney donor transplants. For categorical variables, we presented the data in the form of numbers and percentages to clearly depict their distribution across the groups. Numerical variables were summarized using mean and standard deviation to provide a straightforward understanding of the data's central tendency and dispersion.

In the United Kingdom, the Kidney Allocation Scheme (UK‐KAS) summarizes donor–recipient HLA compatibility using a four‐level mismatch framework that incorporates mismatches across the HLA‐A, HLA‐B, and HLA‐DR loci. Level 1 represents a complete match (000) at all three loci, while Level 2 encompasses minimal mismatch configurations such as 0 DR + 0/1 B or 1 DR + 0 B. Level 3 denotes intermediate mismatch patterns (for example, 0 DR + 2 B or 1 DR + 1 B), and Level 4 corresponds to the highest degree of incompatibility, including combinations such as 1 DR + 2 B or 2 DR. These four levels are embedded in the UK‐KAS points‐based allocation algorithm to balance immunological compatibility with equity and waiting‐time considerations. The framework, introduced in the 2006 national allocation revision, has since been retained and remains integral to organ matching within the UK. In this study, we analyzed these established categories to evaluate their continued clinical relevance in the context of contemporary immunosuppressive regimens.

#### 2.3.2 Analysis for Graft Survival

We defined patient survival as a competing risk to graft survival, and hence, we performed a competing risk Cox regression, where patient survival was a competing risk to graft survival. We also assessed graft survival across HLA mismatch groups defined by UK KAS allocation levels (Levels 1–4), to determine whether these composite groupings retain clinical relevance in contemporary practice.

#### 2.3.3 Univariate Competing Risk Cox Regression Analysis

Initially, a univariate competing risk Cox regression analysis was conducted for each variable to determine its individual impact on graft survival. Variables that showed a significant association with the outcome (*p* value < 0.05) were considered for further analysis in the multivariate model. This step was crucial to identify potential predictors for graft survival without the confounding effects of other variables.

#### 2.3.4 Multivariate Competing Risk Cox Regression Analysis

Significant variables from the univariate analysis were included in a multivariate competing risk Cox regression model. To refine the model and identify the most impactful predictors, we employed a backward stepwise method. This technique iteratively removes the least significant variable until the best fit model is achieved, based on the Akaike information criterion or a similar measure [11]. Importantly, variables representing HLA mismatches (HLA‐A, B, DR, and DQ) were kept in the model a priori, given their known biological relevance to transplant outcomes. Due to presence of linkage disequilibrium between the HLA‐DQ and the HLA‐DR mismatch, we did sensitivity analysis by running the multivariate regression on the patients who had zero HLA‐DR mismatch.

#### 2.3.5 Handling Missing Data

Recognizing the potential bias introduced by missing data, we applied a simple imputation technique to handle missing values. This approach helped maintain the robustness of the analysis by allowing the inclusion of all sampled cases in the regression model, thus avoiding the loss of valuable information.

#### 2.3.6 Analysis for Acute Rejection

A univariate logistic regression analysis was conducted. Variables with a *p* value less than 0.05 were retained for the multivariate logistic regression. The different types of HLA mismatches were pre‐specified as covariates in the multivariate model. Simple imputation was employed to address missing data.

#### 2.3.7 Sensitivity Analysis

In order to do sensitivity analysis for the results of the competing risk cox regression, we reiterated the analysis after truncation at 5 and 10 years post‐transplant.

#### 2.3.8 Statistical Software

All statistical analyses were performed using [STATA package‐17], which is well‐regarded for its reliability and wide range of capabilities in handling complex survival analysis [12].

## 3 Results

The number of patients included in our study was 26 054. Out of these, 209 patients were excluded due to missing data about any type of HLA mismatch, and 642 patients were excluded due to primary non‐functioning kidney and 109 missing data about graft survival. Our analysis included 25 094 patients who received transplants from deceased kidney donors. The baseline characteristics for our cohort are shown in Table 1. Median follow‐up time was 5 years post‐transplant.

**TABLE 1:** Baseline characteristics of the cohort.

Variable	Value
Recipient factors	
Age, years, mean (SD)	50.9 (13.4)
BMI, mean (SD)	26.6 (4.7); missing = 5357 (21.3%)
Diabetes, *n* (%)	4253 (16.9%)
CMV‐positive, *n* (%)	12 751 (53.8%); missing = 1398 (5.6%)
On dialysis at transplantation, *n* (%)	21 345 (85.2%); missing = 28 (<0.1%)
Male sex, *n* (%)	15 638 (62.3%)
Ethnicity, *n* (%)	White 18 418 (73.4); Asian 3654 (14.6); Black 2056 (8.2); Other 915 (3.6); Not reported 53 (0.2)
Donor factors	
Age, years, mean (SD)	49.2 (16.2); missing = 1 (<0.01%)
Extended‐criteria donor, *n* (%)	8998 (35.9%)
CMV‐positive, *n* (%)	12 152 (48.4%); missing = 272 (1.1%)
Male sex, *n* (%)	13 626 (54.3%)
Ethnicity, *n* (%)	White 23 537 (93.8); Asian 537 (2.1); Black 318 (1.3); Other 675 (2.7); Not reported 117 (0.5)
Hypertension, *n* (%)	6560 (26.1%); missing = 522 (2.1%)
Donor Risk Index, mean (SD)	1.22 (0.41); missing = 1107 (4.4%)
Transplant factors	
Cold ischemia time, h, mean (SD)	14.4 (4.9); missing = 248 (1.0%)
Delayed graft function, *n* (%)	6031 (24.0%); missing = 1846 (7.4%)
HLA mismatches, *n* (%)	
HLA‐A: 0/1/2	5067 (20.2)/12 116 (48.3)/7913 (31.5)
HLA‐B: 0/1/2	4027 (16.1)/16 686 (66.5)/4383 (17.5)
HLA‐DR: 0/1/2	11 174 (44.5)/11 979 (47.7)/1943 (7.7)
HLA‐DQ: 0/1/2	10 040 (40.0)/12 450 (49.6)/2606 (10.4)

### 3.1 Impact of Individual HLA Locus Mismatches on Graft Survival

**FIGURE 1:**
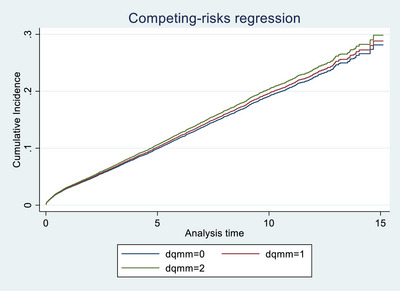
HLA DQ mismatch among deceased transplant patients.

**FIGURE 2:**
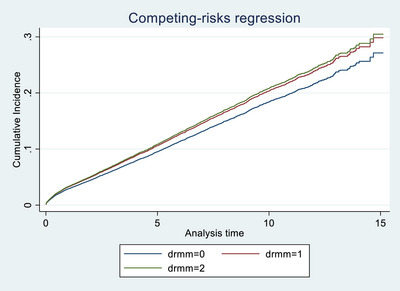
HLA DR mismatch among deceased transplant patients.

**FIGURE 3:**
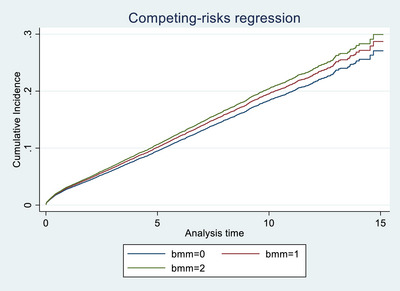
HLA B mismatch among deceased transplant patients.

**FIGURE 4:**
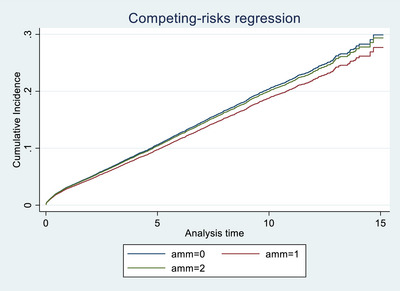
HLA A mismatch among deceased transplant patients.

In the overall cohort, only mismatching at the **HLA‐DR locus** was significantly associated with graft failure. A single HLA‐DR mismatch was associated with increased risk (SHR 1.119, 95% CI 1.035–1.211, *p* = 0.005), while two mismatches showed a non‐significant trend in the same direction (SHR 1.148, *p* = 0.070), possibly due to smaller sample size. In contrast, mismatches at the **HLA‐A**, **HLA‐B**, and **HLA‐DQ** loci were **not significantly associated** with graft outcomes. These findings are illustrated in Figures 1, 2, 3, 4 and Table 2.

**TABLE 2:** Multivariate cox regression for the deceased kidney donor transplant.

Variable	Hazard ratio	*p* value	95% Confidence Interval
Recipient age	0.981	<0.001	0.978–0.984
Dialysis at transplantation (no/yes)	1.284	<0.001	1.151–1.432
CRF	1.003	<0.001	1.002–1.004
HLA DR mismatch			
HLA DR[1]	1.141	0.001	1.053–1.236
HLA DR[2]	1.186	0.026	1.020–1.378
HLA DQ mismatch			
HLA DQ[1]	1.031	0.452	0.953–1.115
HLA DQ[2]	1.080	0.242	0.950–1.227
HLA A mismatch			
HLA A[1]	0.911	0.071	0.824–1.008
HLA A[2]	0.975	0.648	0.876–1.086
HLA B mismatch			
HLA B[1]	1.082	0.163	0.969–1.209
HLA B[2]	1.146	0.047	1.002–1.310
Recipient CMV (no/yes)	1.022	0.542	0.952–1.097
Donor CMV (no/yes)	1.077	0.032	1.007–1.152
Donor age	1.014	<0.001	1.010–1.018
Donor sex (M)	1.010	0.760	0.945–1.080
Recipient ethnicity			
(Asian)	0.934	0.191	0.843–1.035
(Black)	1.393	<0.001	1.246–1.557
(Other)	0.878	0.215	0.714–1.079
(Unknown)	0.512	0.045	0.266–0.986
Donor ethnicity			
(Asian)	1.425	0.001	1.161–1.749
(Black)	1.058	0.697	0.796–1.408
(Other)	1.104	0.424	0.866–1.408
(Unknown)	0.770	0.434	0.400–1.482
Donor history of hypertension (no/yes)	1.114	0.031	1.010–1.229
Extended criteria donor (no/yes)	1.169	0.011	1.037–1.317
Cold ischemia time (hours)	1.008	0.026	1.001–1.014
donor_risk_index	1.212	0.033	1.016–1.445
Delayed graft function (no/yes)	1.422	<0.001	1.323–1.527

A sensitivity analysis restricted to patients with zero HLA‐DR mismatches confirmed that HLA‐DQ mismatches did not independently predict graft failure (Table S1).

### 3.2 Subgroup Analysis Among Different Ethnic Groups

In this competing risk analysis, significant associations were identified across various mismatch categories within different ethnic subgroups. Among the Black subgroup, the analysis revealed that donor‐recipient mismatches in the DR category were associated with reduced sub‐distribution hazard ratios (SHRs), specifically, a SHR of 0.693 (95% CI: 0.432, 1.113) for Level 2, however, it did not reach statistical significance (*p* = 0.129). Conversely, in the Asian subgroup, the Level 1 DR mismatch was significantly associated with an increased SHR of 1.407 (95% CI: 1.138, 1.739, *p* = 0.002). The A and B mismatch categories showed no significant impacts on the hazard ratios across both ethnic groups, with *p* values consistently exceeding 0.4. Results of the competing risk analysis among the Black and Asian subgroups are shown in Tables S2 and S3, respectively. Ethnic differences in HLA mismatch distribution were observed. Details are shown in Table 3. Formal statistical comparison confirmed that mismatch frequencies differed significantly by recipient ethnicity. For each locus (HLA‐A, ‐B, ‐DR, and ‐DQ), chi‐square testing demonstrated highly significant variation among ethnic groups (*p* < 0.001), validating that the observed disparities in mismatch exposure were statistically robust. Black recipients had the highest proportion of two‐level mismatches at the HLA‐DQ locus (14.83%), compared to Asian (10.68%) and White (9.56%) groups. Additionally, Black (44.65%) and Asian (41.25%) recipients had higher rates of two‐level HLA‐A mismatches than White recipients (27.85%). Similarly, optimal HLA‐B matching (0 mismatches) was notably lower among Asian (7.83%) and Black (8.75%) recipients compared to Whites (18.82%). These differences highlight an ethnic variation in HLA mismatch exposure across kidney transplant recipients.

**TABLE 3:** Distribution of different HLA mismatches across the ethnic groups.

HLA locus	Mismatch category	White (%)	Asian (%)	Black (%)	*p* value (*χ* ^2^ test)
HLA‐A	0 mismatch	23.59	38.76	8.75	
	1 mismatch	48.55	48.18	46.60	
	2 mismatch	27.85	41.25	44.65	***p* < 1 × 10^−^¹¹¹** (*χ* ^2^ = 522.7, df = 4)
HLA‐B	0 mismatch	18.82	7.83	8.75	
	1 mismatch	64.80	70.54	72.42	
	2 mismatch	16.38	21.63	18.82	***p* ≈ 1.2 × 10^−^⁸¹** (*χ* ^2^ = 383.1, df = 4)
HLA‐DR	0 mismatch	46.03	41.58	38.62	
	1 mismatch	46.41	50.83	55.01	
	2 mismatch	7.56	7.58	14.83	***p* ≈ 4.2 × 10^−^^3^ ^3^** (*χ* ^2^ = 157.9, df = 4)
HLA‐DQ	0 mismatch	41.70	38.76	30.16	
	1 mismatch	48.74	50.56	55.01	
	2 mismatch	9.56	10.68	14.83	***p* ≈ 2.6 × 10^−^^2^⁷** (*χ* ^2^ = 130.8, df = 4)

## 4 Analysis of the HLA Groups in the UK‐KAS

We evaluated the impact of different HLA levels on graft survival within the UK's kidney allocation system, which categorizes HLA mismatches into four distinct levels. These levels range from HLA Level 1, representing a perfect match (000 mismatch), to HLA Level 4, which indicates the highest degree of mismatch (including combinations such as [1 DR and 2 B] or [2 DR]).

Our multivariate competing risk regression analysis revisited these groupings to assess their differential impacts on graft survival. We observed that transitioning from HLA Levels 1 to 2 does not significantly alter graft survival outcomes, as indicated by a sub‐distribution hazard ratio of 0.973 (95% CI: 0.863, 1.097, *p* = 0.656). This suggests that moving to a Level 2 mismatch, which includes configurations like [0 DR and 0/1 B] or [1 DR and 0 B], does not substantially increase the risk of graft failure compared to a perfect match.

**FIGURE 5:**
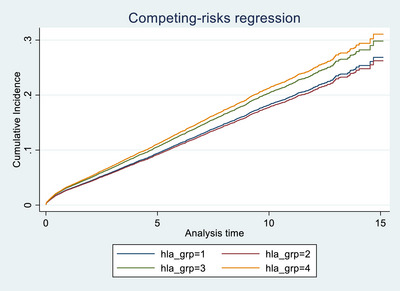
HLA groups among deceased transplant patients.

However, a significant escalation in risk was noted with higher mismatch levels. Specifically, HLA Level 3 mismatches, which involve [0 DR and 2 B] or [1 DR and 1 B], are associated with a notable increase in the risk of graft failure. The sub‐distribution hazard ratio for Level 3 mismatches is 1.132 (95% CI: 1.010, 1.269, *p* = 0.033), representing a 13% increase in risk relative to Level 1. The most pronounced risk increase is associated with HLA Level 4 mismatches, where the hazard ratio escalates to 1.190 (95% CI: 1.037, 1.366, *p* = 0.013). This level of mismatch indicates a 19% higher risk of graft failure compared to the baseline, underscoring a significant impact on graft survival as the mismatch level increases. Details of the results of the sensitivity analysis are shown in Table S4. Figure 5 represents the cumulative incidence ratio.

These findings highlight the progressive risk associated with escalating HLA mismatch levels in kidney transplantation and underscore the critical importance of stringent HLA matching to optimize transplant outcomes. The results support the need for meticulous attention to HLA compatibility in the organ‐matching process, emphasizing that higher mismatch levels can substantially compromise graft survival.

### 4.1 Relationship Between Acute Rejection Episodes and HLA Mismatches

#### 4.1.1 Deceased Kidney Donor Transplant

Acute rejection episodes were under‐reported in the dataset, where it was only available for 80% of the patients. Despite this limitation, the logistic regression analysis among the deceased kidney donor transplants revealed significant associations between HLA‐DQ mismatch and acute rejection. Specifically, DQ mismatches at Levels 1 and 2 were significantly associated with increased risks of acute rejection. For DQ mismatch Level 1, the odds ratio (OR) was 1.202 (95% CI: 1.064–1.358, *p* = 0.003), and for Level 2, the OR increased to 1.464 (95% CI: 1.215–1.764, *p* < 0.001). This relationship remained significant even when we repeated the analysis among those with HLA‐DR zero mismatch, when HLA‐DQ mismatch Level 1 had an OR = 1.36 (*p* value < 0.001), and for Level 2, the OR increased to 1.80 (*p* value = 0.003). Interestingly, HLA DR, B and A mismatch were not associated with higher risk of acute rejection. However, when we performed a subgroup analysis among those with zero HLA DQ mismatch, the HLA DR mismatch was associated with significantly higher risk of early acute rejection (*p* < 0.001). Details of the results are shown in Table S5. Comparison between those with and without missing data about acute rejection is shown in Table S6.

#### 4.1.2 Results of the Sensitivity Analysis

In the competing risks model truncated at 5 years, a moderate HLA‐DR mismatch (DR mismatch = 1) was associated with a 12% increase in the subdistribution hazard of graft failure (sdHR 1.12, 95% CI 1.02–1.24; *p* = 0.019). A single HLA‐A mismatch (A mismatch = 1) was associated with a 12% reduction in hazard compared with no mismatch (sdHR 0.88, 95% CI 0.78–1.00; *p* = 0.043). Low‐risk borderline HLA‐B mismatch (B mismatch = 1) and high‐risk borderline mismatch (B mismatch = 2) were also independently predictive of increased failure risk, with sdHRs of 1.16 (95% CI 1.01–1.33; *p* = 0.033) and 1.24 (95% CI 1.05–1.46; *p* = 0.011), respectively. However, when follow‐up was extended to 10 years, only HLA‐DR mismatches remained statistically significant: a moderate mismatch (DR mismatch = 1) was associated with a 13% higher hazard (sdHR 1.13, 95% CI 1.04–1.23; *p* = 0.003), and a high mismatch (DR mismatch = 2) with a 17% higher hazard (sdHR 1.17, 95% CI 1.00–1.36; *p* = 0.044). The effects of HLA‐A and HLA‐B mismatches were no longer significant at the 10‐year timepoint. Table 4 summarizes the results of the sensitivity analysis.

**TABLE 4:** Sensitivity analysis by truncating at 5 and 10 years.

Variable	Category	5‐year truncated HR (95% CI)	*p* value	10‐year truncated HR (95% CI)	*p* value
DR mismatch	1	1.12 (1.02–1.24)	0.019	1.13 (1.04–1.23)	0.003
	2	0.90 (0.74–1.10)	0.319	1.17 (1.00–1.36)	0.044
DQ mismatch	1	1.05 (0.96–1.16)	0.294	1.02 (0.95–1.11)	0.554
	2	1.09 (0.93–1.28)	0.305	1.08 (0.94–1.23)	0.278
A mismatch	1	0.88 (0.78–1.00)	0.043	0.91 (0.82–1.00)	0.058
	2	0.96 (0.84–1.09)	0.534	0.96 (0.86–1.07)	0.493
B mismatch	1	1.16 (1.01–1.33)	0.033	1.07 (0.95–1.20)	0.250
	2	1.24 (1.05–1.46)	0.011	1.14 (0.99–1.30)	0.059

## 5 Discussion

In this large UK registry analysis of over 25 000 deceased donor kidney transplants, we found that the clinical benefit of HLA‐DR matching is not uniform across ethnic groups. Although a single DR mismatch was associated with increased risk of graft failure in the overall cohort, this benefit did not extend clearly to Black recipients. Our findings raise important concerns about the equity of current allocation policies, particularly regarding the use of low‐resolution HLA matching to guide prioritization.

Our subgroup analysis suggests that DR mismatching may offer limited, if any, survival benefit in Black patients, while DR matching may confer advantages in Asian recipients. This likely reflects the inadequacy of low‐resolution typing to capture immunologically meaningful differences across ethnically discordant donor‐recipient pairs. Previous work by Bekbolsynov et al. has highlighted that apparent DR “matches” based on low‐resolution types may conceal true mismatches at the molecular level, particularly when donor and recipient are from different ethnic backgrounds [13]. Our findings reinforce this concern by demonstrating that DR matching does not predict better graft outcomes in Black patients, despite its prioritization in UK allocation scoring.

Importantly, our analysis also demonstrates ethnic disparities in the distribution of HLA mismatches, with Black recipients more likely to receive poorly matched grafts. This structural mismatch imbalance, combined with lack of benefit from DR matching, suggests that the current allocation framework may inadvertently delay access to transplantation for Black patients without providing compensatory clinical benefit. This echoes concerns raised decades ago about the potential for HLA‐based allocation to disadvantage ethnic minorities [14]. Our data now provide contemporary evidence that such disparities persist and require reconsideration in light of modern immunosuppression.

Additionally, we evaluated the current UK Kidney Allocation Scheme's grouping of HLA mismatch levels. We found that the four‐level classification does differentiate graft survival risk: higher mismatch levels (particularly Level 4) were associated with progressively worse outcomes. This supports the internal consistency of the UK‐KAS grouping, though the benefits appear modest and likely concentrated among subgroups such as White recipients. These results underscore the need for more nuanced stratification—ideally incorporating both immunologic relevance and ethnic‐specific performance.

The relationship between HLA mismatches and acute rejection episodes further complicates the picture. While DR mismatches were not independently associated with early rejection, DQ mismatches showed a strong and consistent relationship with acute rejection risk—even among DR‐matched patients. This finding supports growing evidence that DQ antigens may play an underappreciated role in alloimmunity. However, DQ mismatches did not independently predict long‐term graft failure in our cohort, suggesting that while they may trigger early rejection, they may not have a sustained impact on allograft loss [15, 16, 17].

In light of our findings highlighting ethnic disparities in HLA mismatch exposure and clinical outcomes, we recommend specific modifications to the UK Kidney Allocation Scheme (KAS). This includes introducing ethnicity‐specific mismatch scoring to prioritize HLA‐DR matching for Asian recipients, reducing exposure to high‐risk (two‐level) HLA‐A and B mismatches among Asian and Black recipients, and explicitly addressing the higher incidence of HLA‐DQ mismatches among Black recipients. Although DR matching remains a core feature of UK‐KAS, our data show *no statistically significant graft‐survival benefit* from DR compatibility in Black recipients. We believe this reflects the limitations of the current low‐resolution HLA typing—which can mask true molecular mismatches in ethnically discordant pairs—and the unintended consequence that Black candidates may wait longer for DR “matches” that do not translate into clinical gain. Thus, it is essential to highlight this finding to avoid equity harms, rather than to suggest that matching per se is without value for everyone. Furthermore, we propose mandatory annual ethnicity‐based auditing of mismatch and outcome data to ensure continued monitoring and timely intervention. Implementing these targeted, ethnicity‐specific measures could significantly reduce structural inequalities and enhance graft outcomes across all transplant recipients. Detailed recommendations for the KAS are shown in Table 5 [18, 19]. To translate our subgroup findings into actionable policy, we developed Table 5 to outline targeted adjustments across all HLA loci—A, B, DQ, and DR—using the same low/intermediate resolution data that underlies UK‐KAS. By applying uniform typing limitations to inform ethnicity‐specific weighting (e.g., penalizing two‐level mismatches more heavily in minority recipients, prioritizing DR for those groups where it remains beneficial, and adding regular audits), these recommendations do not assume fine resolution at any locus but instead use the shared data constraints to correct identified equity harms (Table 5). Table 5 is designed for the entire transplant cohort—not just Black recipients—and provides a template for how UK KAS could operationalize ethnicity‐specific HLA scoring. For example, the existing point system could be modified via simple weighting factors (e.g., multiplying mismatch penalties by 1.2 for minority groups at certain loci) and automated within the allocation software to ensure seamless integration. The recommendations summarized in Table 5 are intended as conceptual examples derived from our subgroup findings rather than as prescriptive policy changes. Their purpose is to highlight possible directions for improving equity within the existing low‐resolution framework. Future modeling studies and simulation analyses are needed to test the feasibility and clinical implications of these proposed adjustments before implementation. Our study has several strengths. It leverages a large, nationally representative dataset with extended follow‐up and applies a competing‐risks framework appropriate for transplant outcomes. Assessing both individual HLA loci and grouped mismatch levels, it provides a comprehensive evaluation of the UK Kidney Allocation Scheme's performance in current clinical practice.

**TABLE 5:** Proposed ethnicity‐specific adjustments to HLA mismatch scoring policies under low‐resolution typing constraints. All recommendations are based on low/intermediate‐resolution HLA typing data as used in UK‐KAS. Ethnicity‐specific adjustments leverage these uniform data constraints—drawing on subgroup analyses (Table 3) that showed limited graft‐survival benefit of DR matching in Black recipients—to introduce targeted weighting across HLA‐A, ‐B, ‐DQ, and ‐DR, prioritizing loci with higher immunogenic impact and correcting identified equity harms.

Policy area	Current policy	Recommended change	Example implementation
Ethnicity‐specific adjustment of HLA mismatch scoring	Mismatch points assigned equally across all ethnicities.	Introduce ethnicity‐specific weighting, penalizing two‐level mismatches at critical loci (DQ, DR, A, B) for minority groups.	Apply additional penalty points (–200 points) for two‐level mismatches at HLA‐A/B loci and (–150 points) for DQ mismatches in minority recipients.
Prioritization of DR matching for Asian recipients	No ethnicity‐specific prioritization at HLA‐DR.	Add bonus points specifically for optimal DR matches in Asian recipients.	Award 200–500 additional points for zero or one‐level DR mismatches specifically for Asian recipients.
Reduction of two‐level mismatches at HLA‐A and HLA‐B loci	No ethnicity‐specific constraints on HLA‐A/B mismatches.	Implement penalty points or constraints specifically to reduce two‐level HLA‐A/B mismatches for Black and Asian recipients.	Assign additional penalty points (–200) for two‐level mismatches at HLA‐A/B loci for Black and Asian recipients.
Enhanced matching for HLA‐DQ mismatches	HLA‐DQ mismatches scored equally for all recipients.	Increase penalties specifically for two‐level DQ mismatches among Black recipients.	Assign additional penalty points (–150) for two‐level DQ mismatches specifically for Black recipients.
Mandatory ethnicity‐based auditing	Regular reviews conducted without explicit ethnicity focus.	Mandate annual ethnicity‐based audits of mismatch distribution and transplant outcomes.	Annual NHSBT reports explicitly stratified by ethnicity, guiding timely policy revisions.

While HLA typing in this study is of low/intermediate resolution, it reflects the data presently used in the UK allocation system. This alignment ensures that our findings are directly applicable to real‐world policy and offer a realistic assessment of how the current framework functions, particularly in relation to ethnic disparities in mismatch exposure. Our aim was not to critique the quality of the underlying data, but to identify opportunities for improvement within the constraints of existing practice. By focusing on the stratification and weighting currently employed, our analysis yields actionable insights that could be implemented immediately to enhance equity and outcomes in kidney allocation.

We acknowledge several limitations. Ethnicity in the UK Transplant Registry is self‐reported and may not accurately represent genetic ancestry, which could lead to minor misclassification and attenuate or obscure true biological differences in HLA effects across ethnic groups. Another major limitation is the absence of data on de novo donor‐specific antibodies (DSA), particularly class II (DQ) antibodies, which are known mediators of late antibody‐driven graft loss. Because the registry does not routinely capture DSA status or monitoring data, we were unable to account for this mechanism directly—potentially explaining the discordance between early rejection risk and long‐term graft failure observed for DQ mismatches.

Emerging molecular approaches, such as eplet‐level typing of HLA class II loci (including DRβ and DQα/β), have demonstrated greater precision in predicting antibody formation and graft outcomes than conventional low‐resolution matching. Although such data are not yet available within the UK Transplant Registry, integrating eplet‐based metrics into future allocation algorithms could enhance both immunological precision and equity. Ultimately, the registry's current limitations—namely, the absence of DSA and eplet‐level data—preclude deeper analyses of molecular mismatch or antibody‐mediated mechanisms, but they also highlight the potential value of incorporating these variables in future national datasets.

In conclusion, our study demonstrates that HLA‐DR mismatching is associated with poorer graft survival overall, but this association does not appear consistent across all ethnic groups—most notably among Black recipients, for whom DR matching seems to confer limited clinical benefit. These findings raise important questions about the equity of current UK allocation practices, which prioritize DR compatibility without considering its variable predictive value across populations.

While the existing HLA grouping system used in the UK Kidney Allocation Scheme—comprising four mismatch levels that integrate HLA‐A, HLA‐B, and HLA‐DR loci—does stratify graft survival risk, its clinical utility may be constrained by low‐resolution typing and lack of ethnicity‐specific calibration. Larger, prospectively validated datasets, incorporating high‐resolution molecular and antibody data, are needed to confirm these findings and evaluate whether the observed patterns justify adjustments to allocation weighting or refinement of the current four‐level HLA grouping framework.

Ultimately, future policy refinements should aim to ensure that strategies to enhance HLA compatibility do not inadvertently disadvantage patients for whom current matching methods offer limited or no tangible benefit. Our study provides evidence to inform this discussion and highlights the need for ongoing research to optimize both equity and immunological precision in organ allocation.

## Funding

The authors have nothing to report.

## Conflicts of Interest

The authors declare no conflicts of interest.

## Supporting information




**Supporting Table S1:** Comparison between those with missing data about acute rejection versus those with no missing data among deceased kidney donor transplants.
**Supporting Table S2:** Multivariate cox regression for the deceased kidney donor transplant in subgroup of patients with HLA DR = 0.
**Supporting Table S3:** Results of the HLA groups in the multivariate cox regression among the deceased kidney donor transplants.
**Supporting Table S4:** Multivariate competing risk cox regression for the deceased kidney donor transplant in subgroup of patients with black ethnicity.
**Supporting Table S5:** Multivariate competing risk cox regression for the deceased kidney donor transplant in subgroup of patients with Asian ethnicity.
**Supporting Table S6:** Results of the HLA mismatches and its relationship with acute rejection among the deceased kidney donor transplants using the multivariate logistic regression.

## Data Availability

The data that support the findings of this study are available from UKTR. Restrictions apply to the availability of these data, which were used under license for this study. Data are available from the author(s) with the permission of UKTR.
