# Supplementary material for: HLA‐DR Matching in Kidney Transplantation: Ethnic Disparities in Clinical Benefit and Policy Implications From a UK Registry Analysis
Source: Clin Transplant. 2026 Jan 6;40(1):e70429. doi: 10.1111/ctr.70429 (PMC12771550; doi:10.1111/ctr.70429)
Supplement: Supplementary file 1 — Supporting Table S1: Comparison between those with missing data about acute rejection versus those with no missing data among deceased kidney donor transplants. Supporting Table S2: Multivariate cox regression for the deceased kidney donor transplant in subgroup of patients with HLA DR = 0. Supporting Table S3: Results of the HLA groups in the multivariate cox regression among the deceased kidney donor transplants. Supporting Table S4: Multivariate competing risk cox regression for the deceased kidney donor transplant in subgroup of patients with black ethnicity. Supporting Table S5: Multivariate competing risk cox regression for the deceased kidney donor transplant in subgroup of patients with Asian ethnicity. Supporting Table S6: Results of the HLA mismatches and its relationship with acute rejection among the deceased kidney donor transplants using the multivariate logistic regression. [file CTR-40-e70429-s001.docx]

|  | Missing data about acute rejection (n=5573) | No missing data about acute rejection (n=19,523) |
| --- | --- | --- |
| **Recipient factors:** |  |  |
| -Recipient age: mean (standard deviation) | 51.48358 (13.4115) | 50.7105 (13.38632 ) |
| -Recipient BMI: mean (standard deviation) | 26.84386 (4.782127) | 26.52578 (4.72765) |
| -Recipient Diabetes:  -No : n (%)  -Yes: n (%) | 4,526 (81.21%)  1,047 (18.79%) | 16,317 (83.58%)  3,206 (16.42%) |
| -Recipient CMV:  -No : n (%)  -Yes: n (%)  -Missing data : n (%) | 2,399 ( 43.05%)  2,927 (52.52%)  247 (4.43%) | 8,548 (43.78%)  9,824 (50.32%)  1,151 (5.90%) |
| -Dialysis at time of transplantation:  -No : n (%)  -Yes: n (%)  -Missing data : n (%) | 882 (15.83%)  4,687 (84.10%)  4 (0.07%) | 2,841 (14.55%)  16,658 (85.33%)  24 (0.12%) |
| -Recipient sex:  -Male : n (%)  -Female: n (%) | 3,479 (62.43%)  2,094 (37.57%) | 12,159 (62.28%)  7,364 (37.72%) |
| -Recipient ethnicity:  -White : n (%)  -Black: n (%)  -Asian : n (%)  -Other : n (%)  -Not reported: n(%) | 3,995 (71.68%)  453 (8.13%)  850 (15.25%)  214 (3.84%)  61 (1.09%) | 14,423 (73.88%)  1,603 (8.21%)  2,804 (14.36%)  572 (2.93%)  121 (0.62%) |
|  |  |  |
| **Donor factors:** |  |  |
| -Donor age: mean (standard deviation) | 49.47523 (16.08013) | 49.17292 (16.25416) |
| -Extended criteria donor:  -No : n (%)  -Yes: n (%)  -Missing data : n (%) | 3,555 (63.79 %)  2,017 (36.19%)  1 (0.02%) | 12,542 ( 64.24%) 6,981 (35.76%)  none |
| -Donor CMV:  -No : n (%)  -Yes: n (%)  -Missing data : n (%) | 2,907 (52.16 %)  2,615 (46.92%)  51 (0.92%) | 9,765 (50.02%)  9,537 ( 48.85%)  221 ( 1.13%) |
| -Donor sex:  -Male : n (%)  -Female: n (%)  -Missing data : n (%) | 3,078 (55.23%)  2,495 (44.77%)  none | 10,548 (54.03%)  8,975 ( 45.97%) none |
| -Donor ethnicity:  -White : n (%)  -Black: n (%)  -Asian : n (%)  -Other : n (%)  -Not reported: n(%) | 5,148 ( 92.37% )  143 (2.57 % )  78 (1.40 % )  154 (2.76% )  50 ( 0.90 % ) | 18,389 (94.19 % )  394 (2.02 % )  240 (1.23 % )  404 (2.07 %)  96 ( 0.49 % ) |
| -Donor hypertension:  -No : n (%)  -Yes: n (%)  -Missing data : n (%) | 4,042 ( 72.53%)  1,432 (25.70%)  99 (1.78%) | 13,972 ( 71.57%)  5,128 (26.27%)  423 (2.17%) |
| Donor risk index (for deceased transplants only):  mean (standard deviation): | 1.211383 (0 .424434) | 1.225345 (0.4076496) |
|  |  |  |
| **Transplant factors:** |  |  |
| -CRF: mean (standard deviation) | 22.21335 (34.40752) | 21.23121 ( 33.74397 ) |
| -Cold ischemia time: mean (standard deviation) | 13.46703 (4.385316 ) | 14.67091 (5.078883) |
| -Delayed graft function:  -No : n (%)  -Yes: n (%)  -Missing data : n (%) | 3,026 (54.30%)  1,005 (18.03%)  1,542 (27.67%) | 14,193 (72.70%)  5,026 (25.74%)  304 (1.56%) |
| HLA DQ mismatch:  0 : n (%)  1 :n (%)  2: n (%)  Missing data : n(%) | 2,183 (39.17 %)  2,784 (49.96 %)  606 (10.87%) | 7,857 (40.24%)  9,666 (49.51%)  2,000 (10.24%) |
| HLA A mismatch:  0 : n (%)  1 :n (%)  2: n (%) | 1,031 (18.50%)  2,812 (50.46%)  1,730 (31.04 %) | 4,036 ( 20.67%)  9,304 (47.66%)  6,183 (31.67%) |
| HLA B mismatch:  0 : n (%)  1 :n (%)  2: n (%) | 835 (14.98 %)  3,627 (65.08 %)  1,111 (19.94 %) | 3,192 (16.35%)  13,059 ( 66.89%)  3,272 (16.76%) |
| HLA DR mismatch:  0 : n (%)  1 :n (%)  2: n (%) | 2,316 (41.56%)  2,782 (49.92%)  475 ( 8.52 %) | 8,858 (45.37%)  9,197 (47.11%)  1,468 (7.52%) |
|  |  |  |
|  |  |  |

Table 1: Comparison between those with missing data about acute rejection versus those with no missing data among deceased kidney donor transplants

| Variable | Hazard Ratio | P-value | 95% Confidence Interval |
| --- | --- | --- | --- |
| HLA DQ |  |  |  |
| HLA DQ (1) | 0.969 | 0.575 | 0.868 - 1.082 |
| HLA DQ (2) | 1.011 | 0.943 | 0.754 - 1.355 |
|  |  |  |  |
| HLA A |  |  |  |
| HLA A (1) | 0.949 | 0.507 | 0.812 - 1.108 |
| HLA A (2) | 0.973 | 0.745 | 0.823 - 1.149 |
|  |  |  |  |
| HLA B |  |  |  |
| HLA B (1) | 0.998 | 0.983 | 0.854 - 1.168 |
| HLA B (2) | 1.192 | 0.075 | 0.982 - 1.446 |

Table 2: Multivariate cox regression for the deceased kidney donor transplant in subgroup of patients with HLA DR=0

| Variable | Hazard Ratio | P-Value | 95% Confidence Interval |
| --- | --- | --- | --- |
| HLA level 2 (in comparison to level 1) | 0.99 | 0.89 | 0.8771555 to 1.117677 |
| HLA level 3(in comparison to level 1) | 1.16 | 0.009 | 1.04013 to 1.310912 |
| HLA level 4 (in comparison to level 1) | 1.25 | 0.002 | 1.08883 to 1.440154 |

Table 3: Results of the HLA groups in the multivariate cox regression among the deceased kidney donor transplants.

| Mismatch Category | Level | SHR (95% CI) | Std. Err. | Z-Value | P-Value |
| --- | --- | --- | --- | --- | --- |
| DRMM | 1 | 0.889 (0.711, 1.111) | 0.1011 | -1.03 | 0.301 |
| DRMM | 2 | 0.693 (0.432, 1.113) | 0.1674 | -1.52 | 0.129 |
| DQMM | 1 | 1.022 (0.811, 1.288) | 0.1206 | 0.18 | 0.854 |
| DQMM | 2 | 1.214 (0.870, 1.694) | 0.2063 | 1.14 | 0.254 |
| AMM | 1 | 1.064 (0.720, 1.572) | 0.2118 | 0.31 | 0.756 |
| AMM | 2 | 1.165 (0.793, 1.712) | 0.2287 | 0.78 | 0.436 |
| BMM | 1 | 0.950 (0.642, 1.407) | 0.1902 | -0.26 | 0.798 |
| BMM | 2 | 0.873 (0.556, 1.371) | 0.2008 | -0.59 | 0.556 |

Table 4: Multivariate competing risk cox regression for the deceased kidney donor transplant in subgroup of patients with black ethnicity.

| Mismatch Category | Level | SHR (95% CI) | Std. Err. | Z-Value | P-Value |
| --- | --- | --- | --- | --- | --- |
| DRMM | 1 | 1.407 (1.138, 1.739) | 0.1520 | 3.16 | 0.002 |
| DRMM | 2 | 1.025 (0.666, 1.577) | 0.2253 | 0.11 | 0.910 |
| DQMM | 1 | 1.147 (0.928, 1.418) | 0.1241 | 1.27 | 0.204 |
| DQMM | 2 | 1.137 (0.803, 1.610) | 0.2017 | 0.72 | 0.470 |
| AMM | 1 | 0.879 (0.642, 1.205) | 0.1412 | -0.80 | 0.423 |
| AMM | 2 | 1.057 (0.772, 1.446) | 0.1691 | 0.34 | 0.731 |
| BMM | 1 | 0.871 (0.625, 1.214) | 0.1476 | -0.82 | 0.414 |
| BMM | 2 | 1.123 (0.775, 1.628) | 0.2128 | 0.61 | 0.540 |

Table 5: Multivariate competing risk cox regression for the deceased kidney donor transplant in subgroup of patients with Asian ethnicity.

| Variable | Odds Ratio | 95% CI | P-value |
| --- | --- | --- | --- |
| Recipient age | 0.980 | 0.976 - 0.985 | <0.001 |
| CRF | 1.003 | 1.001 - 1.004 | 0.001 |
| HLA DR |  |  |  |
| - 1 | 1.074 | 0.951 - 1.213 | 0.251 |
| - 2 | 1.106 | 0.888 - 1.377 | 0.369 |
| HLA DQ |  |  |  |
| - 1 | 1.202 | 1.064 - 1.358 | 0.003 |
| - 2 | 1.464 | 1.215 - 1.764 | <0.001 |
| HLA A |  |  |  |
| - 1 | 0.978 | 0.842 - 1.136 | 0.770 |
| - 2 | 0.919 | 0.782 - 1.081 | 0.309 |
| HLA B |  |  |  |
| - 1 | 1.019 | 0.864 - 1.201 | 0.826 |
| - 2 | 0.978 | 0.799 - 1.197 | 0.826 |
| Donor age | 1.013 | 1.009 - 1.017 | <0.001 |
|  |  |  |  |
| Recipient ethnicity |  |  |  |
| Asian | 0.806 | 0.690 - 0.942 | 0.007 |
| Black | 1.379 | 1.175 - 1.618 | <0.001 |
| Other | 0.843 | 0.617 - 1.152 | 0.283 |
| Unknown | 0.782 | 0.378 - 1.618 | 0.507 |
|  |  |  |  |
| Donor ethnicity |  |  |  |
| Asian | 1.277 | 0.915 - 1.782 | 0.150 |
| Black | 1.670 | 1.151 - 2.422 | 0.007 |
| Other | 1.055 | 0.734 - 1.515 | 0.773 |
| Unknown | 0.622 | 0.250 - 1.548 | 0.308 |
|  |  |  |  |
| Cold ischemia time (hours) | 1.010 | 1.000 - 1.020 | 0.048 |
| Delayed graft function (no/yes) | 2.688 | 2.427 - 2.976 | <0.001 |

Table 6: Results of the HLA mismatches and its relationship with acute rejection among the deceased kidney donor transplants using the multivariate logistic regression.
